# HIF-1*α* Regulates the Progression of Cervical Cancer by Targeting YAP/TAZ

**DOI:** 10.1155/2022/3814809

**Published:** 2022-05-25

**Authors:** Azierguli Abudoukerimu, Axiangu Hasimu, Abudouhabaer Abudoukerimu, Gulijiannaiti Tuerxuntuoheti, Yixin Huang, Jie Wei, Tao Yu, Hong Ma, Delixiati Yimiti

**Affiliations:** ^1^Department of Occupational and Environmental Health, School of Public Health, Xinjiang Medical University, Urumqi, 830017, China; ^2^Department of Microbiology, School of Basic Medical Science, Xinjiang Medical University, Urumqi, 830017, China; ^3^Xinjiang Key Laboratory of Molecular Biology for Endemic Disease, Urumqi, 830017, China; ^4^Department of Pathology, School of Basic Medical Science, Xinjiang Medical University, Urumqi, 830017, China; ^5^Department of Science, Hotan Normal College, Hotan 848000, Xinjiang Uygur Autonomous Regions, China; ^6^Department of Linguistic, Hotan Normal College, Hotan 848000, Xinjiang Uygur Autonomous Regions, China; ^7^Institute of Veterinary Medicine, Xinjiang Academy of Animal Science, Urumqi 830000, Xinjiang Uygur Autonomous Regions, China; ^8^Shandong Institute of Parasitic Diseases, Shandong First Medical University & Shandong Academy of Medical, Jinan 272033, Shandong Province, China

## Abstract

Cervical carcinoma is one of the serious pernicious cancers that influence women's health. Invasion and metastasis are the chief reason of poor prognosis of cervical carcinoma. Hypoxia-inducible factor-1*α* (HIF-1*α*) is a significant regulatory factor of intracellular oxygen supersession, and its expression or increased activity is closely related to the arise and expansion of various human tumors. However, the relationship between HIF-1*α* (hypoxia-inducible factor 1) and Hippo pathway target gene Yes-related protein (YAP) and transcriptional coactivator (TAZ) in cervical carcinoma remains unclear. Here, we studied the clinical correlation of HIF-1*α* and YAP/TAZ expression in normal tissues, cervical intraepithelial neoplasia (CIN), and cervical squamous cell carcinoma (CSCC). In order to analyze the role of HIF-1*α* in CCSC in vitro, SiHa cells with high expression of HIF-1*α* and C33a cells with low expression of HIF-1*α* were screened by detection. After transfection with lentivirus, HIF-1*α* levels were downregulated in SiHa cells and upregulated in C33a Cells, respectively. Then, the expression of HIF-1*α* in transfected cervical cancer cells Siha and C33a was detected by qRT-PCR and Western blot, and the expression of YAP/TAZ was detected in cervical squamous cell carcinoma cells after HIF-1*α* expression was altered. To explore HIF-1*α* role in cell proliferation, invasion, and metastasis, we examined the changes of cell function in cervical cancer cells with HIF-1*α* overexpression and inhibition by MTT assay, wound healing assay, Transwell test, and other cell function tests. At the same time, HIF-1*α* overexpression and HIF-1*α* inhibition cervical cancer cells were transplanted into nude mice, and tumors were isolated from the nude mice, and tumor volume and weight were observed. In conclusion, HIF-1*α* significantly promotes the proliferation, invasion, and migration of cervical carcinoma cells by upregulating YAP/TAZ. In addition, YAP/TAZ, the target gene of Hippo pathway, plays an important role in CCSC cells, pointing out that HIF-1*α* is provided with treatment potential for the treatment of CCSC.

## 1. Introduction

Cervical carcinoma is still one of the major gynecological tumors that seriously imperil women's physical and mental health, only next to breast cancer [1]. According to statistics, about 527000 women in the world have been newly diagnosed with cervical carcinoma, and more than 266000 women have died of cervical cancer. Cervical carcinoma is a pathema of out of control cell division, invasion, and attack of female cervical tissue. Cervical squamous cell carcinoma, as the main histopathological type of cervical cancer, accounts for more than 90% of cervical cancer [2].

Cervical cancer is a major public health problem in China. According to the research, in 2015, there were about 98,900 new cases of cervical cancer in China, accounting for 18.7% of the global incidence rate. The incidence and mortality of cervical cancer have been on the rise. China is the most populous country in the world (about 1 billion 370 million) of whom about 70% live in rural areas and the incidence of cervical cancer in rural women is very high [3]. The incidence rate and mortality rate of cervical squamous cell carcinoma in rural area and the incidence of cervical cancer among rural women are four times the average incidence of cervical cancer in China [4, 5]. According to the survey, among the clinical cases of cancer in rural area of China, the proportion of cervical cancer patients exceeds 20%, of whichrural women may account for 70%, and the mortality rate ranks firstin China, and cervical carcinoma is still a significant health problem for the women of the living in rural areas of China [6]. Since tumor invasion and metastasis are the key to judge tumor prognosis, it is of great significance to explore the invasion and metastasis mechanism of cervical cancer, find effective markers and therapeutic targets to inhibit the invasion and metastasis of cancer cells, and improve the prognosis of cervical cancer; the therapeutic effect and prognosis of patients are of great significance.

Hypoxia is a characteristic of the solid tumor microenvironment which is related to the adverse clinical outcomes of a variety of solid tumors [7–11]. In addition to genetic and environmental factors, tumor microenvironment (TME) also plays an important role in the progression of malignant tumors. TME promotes tumor genesis, progression, metastasis, and deterioration through multiple mechanisms [12–14].

HIF-1*α* is a regulator that can adapt to hypoxic environment and play a major regulatory role in hypoxic environment. HIF-1*α* has been shown to regulate hypoxic gene expression and signal transduction pathways, so it is a potential therapeutic target attracting much attention [15]. The capability of tumor cells to fit to variety in oxygen concentration is crucial to the development of tumors. Hypoxia affects cellular metabolism, thereby affecting abnormal tumor proliferation, migration, and invasion. HIF-1*α* is a focal point of cellular adaptation to hypoxia, affecting cell viability and the expression of specific genes related to cellular metabolism. HIF induces the production of vesicles that promote long-range intercellular communication, for example, promoting tumor angiogenesis [16].

More than 100 genes are activated and upregulated by HIF related to the occurrence of abnormal tumor cell proliferation, abnormal metabolism, tumor invasion and metastasis, tumor treatment resistance, inflammation, and tumor immunity. After the functional study of HIF-1 and HIF-2, it was found that the regulation of target genes in hypoxic cells is mainly controlled by HIF-1 and HIF-2, especially the regulation of angiogenesis and glucose metabolism is the main role of HIF, and this is also the main characteristics of tumors. The tumor angiogenesis driver vascular endothelial growth factor (VEGF) can be regulated by HIF-1 and HIF-2 while also regulating genes involved in glycolysis, such as glucose transporter and lactate dehydrogenase. In addition to this, HIF can reduce the effectiveness of anticancer treatments such as radiotherapy, chemotherapy, and immunotherapy, thereby promoting tumor progression and shortening patient lives [17].

According to reports, HIF-1*α* expression is abnormally elevated in some malignancies, suggesting that HIF-1*α* expression is associated with tumor genesis, progression, and poor prognosis [15].

Yes-related protein (YAP) and transcription coactivator TAZ are the core components of Hippo pathway. Activated YAP/TAZ binds to transcription factors after entering the nucleus and acts on downstream genes related to tumor proliferation. As a transcriptional coactivator, YAP binds to the transcription factor TEAD and promotes the expression of downstream genes, so as to make cells proliferate. TAZ also plays a similar function in tumors. Meanwhile, the involvement of YAP/TAZ in tumor invasion and metastasis has been fully verified. and YAP/TAZ is abnormally upregulated in many human malignant tumors, fibrosis, and cancer [18]. In some cases, YAP and TAZ promote cell growth by activating the transcription program of TEAD family transcription factors [19]. It has been found that in a mouse breast cancer model, specific loss of CCM3 in cancer-associated fibroblasts leads to mutual activation of YAP/TAZ in adjacent tumor cells, resulting in tumor metastasis to distant organs [20]. Activation of YAP/TAZ in pyloric stem cells leads to progressive tumorigenesis and initiates gastric cancer in vivo, and its significance in human gastric cancer has been verified [21]. All the above studies indicate that YAP/TAZ is an essential major regulator in the initiation or growth of most solid tumors and cancers. YAP/TAZ is a sensor for the structural and mechanical properties of the cell microenvironment. Many external and internal cues associated with cancer jointly govern changes in mechanical transduction, inflammation, oncogenic signaling, and regulation of the Hippo pathway. As a transcription coactivator of oncogenes or a transcription corepressor of tumor suppressor genes, YAP/TAZ is involved in the occurrence and development of tumors and the glycolysis process of tumors, which is associated with classical chemotherapy drug resistance and poor prognosis of many cancers [21–23].

In this research, the expression and correlation of HIF-1*α*, YAP, and TAZ in normal cervical tissues, CIN and CSCC tissues, and cervical cancer cells were further detected by in vivo and in vitro assays, so as to further explore the roles of the three in the malignant progression of CSCC.

## 2. Methods

### 2.1. Ethics

Tissue samples used in the experiment were collected from archived paraffin-embedded tissues of outpatients and inpatients undergoing surgery in the Department of Pathology, Changji Hospital of Xinjiang province from January 2016 to July 2019. Clinical data of all cases were complete and reliable. 40 patients with CSCC were aged from 31 to 83 years, with a median age of 55 years. All patients had no preoperative history of radiotherapy, chemotherapy, or immunotherapy. According to the WHO classification standard of 2020, 19 cases were highly differentiated and 21 cases were moderately or poorly differentiated. There were 15 cases with lymph node metastasis and 25 cases without lymph node metastasis. In addition, 58 cases of CIN tissue (32 cases of CIN I and 26 cases of CIN II) and 30 cases of normal adjacent tissues (NATs)were selected. The clinicopathological data of all patients were collected with the consent of the patients and their families, and informed consent was signed. The experiment was approved by the Ethics Committee of the First Affiliated Hospital of Xinjiang Medical University (xjykdxr20211015003).

### 2.2. Immunohistochemistry

Immunohistochemical SP assay was used to detect HIF-1*α*, YAP, and TAZ protein expressions in NATs, CIN, and CSCC. HIF-1*α*, YAP, and TAZ antibodies were from Abcam plc (Cambridge, MA, USA), and SP immunohistochemistry kit and DAB chromogenic agent were from Zhong Shan Goldenbridge Biotechnology Co. Ltd., (China). Paraffin-embedded tissues were sectioned in 3 *μ*m thickness, followed by conventional xylene dewaxing, gradient alcohol hydration, microwave oven antigen repair, and soaked in 3% hydrogen peroxide for ten minutes to remove endogenous peroxidase. They were incubated with anti-HIF-1*α*, YAP, and TAZ antibodies at 4°C overnight. The staining pattern was scored independently by an experienced pathologist. The immunostaining score was estimated semiquantitatively based on staining intensity and distribution. The concentration of HIF-1*α*, YAP, and TAZ antibodies were 1 : 100, 1 : 150, and 1 : 200, respectively. In the next day, biotin-labeled secondary antibody was dropped and incubated at 37°C for 30 minutes. Every step was washed with PBS buffer (pH7.4) for 3 times (10 min). Paraffin-embedded tissues were stained with DAB dye solution, and then the nuclei were counterstained with hematoxylin. Finally, the sections were dehydrated and sealed with neutral gum). In each batch of staining, sections with known positive antibodies were used as positive control, and 0.01 mol/L PBS was used as negative control. HIF-1*α* protein expression products were located in the nucleus, and YAP and TAZ protein expression products were positive in cytoplasm or nucleus when stained with brownish yellow granules. Ten high magnification field positive cells were randomly extracted from each slices, and the average number of positive cells per 100 cells was taken as the positive cell rate. Positive cells with HIF-1*α*, YAP, and TAZ expressions ≥ 25% were considered as positive (+), while cells without staining or positive cell rate < 25% were considered as negative (-).

### 2.3. Cell Culture and Lentiviral Infection

CCSC cell lines SiHa and C33a and cervical squamous epithelial H8 cells were commercially from Shanghai Cell Collection (Shanghai, China). The cells were cultured by DMEM (Hyclone, USA), containing 10% fetal bovine serum (Gibco, New Zealand), 100 U/mL penicillin, and 100 *μ*g/mL streptomycin (Hyclone, USA) maintained at 37°C filled with 5% CO_2_ incubator. Lentiviruses carrying green fluorescent protein shHIF-1*α* (knockdown) and LV-HIF-1*α* (overexpression) were purchased from GENECHEM (Shanghai, China). For infection, SiHa and C33a cells were incubated with lentiviruses (multiplicity of infection (MOI) of 10 and 20, respectively). The cells were transfected in serum-free medium in a 6-well plate. After 72 hours of transfection, the transfection efficiency gradually increased. The cells were transferred to culture medium containing serum for 6-8 days, and then, the transfected cells were sorted out by flow cytometry.

### 2.4. RNA Extraction and qRT-PCR Assay

Total RNA was extracted from SiHa, C33a, and H8 cells by using TRIzol reagent (Invitrogen, Carlsbad, CA, USA) and transcribed into cDNA using reverse transcription PCR using by cDNA Synthesis Kit (TaKaRa, Dalian, China). CDNA was detected using SYBR Green fluorescent quantitative PCR amplification kit and ABI 7500 rapid system (Applied Biosystems, CA, USA) according to the manufacturer's instructions. The human HIF-1*α* primer sequences and glyceraldehyde-3-phosphate dehydrogenase (GAPDH) oligonucleotide primers we used are as follows: HIF-1*α* RT00018656-F-AAGTTCACCTGAGCCTAAT, RT00018656-R-TCTCCAAGTCTAAATCTGTG, and GADPH RT763F-TGACTTCAACAGCGACACCCART763R-CACCCTGTTGCTGTAGCCAAA.

During qRT-PCR, GAPDH was used as internal reference to standardize gene expression levels. 3-5 relative quantization was performed and the average relative change was calculated using the delta-delta cyclic threshold method. All reactions are repeated three times.

### 2.5. Western Blot (WB) Analysis

SiHa C33a H8 cells were washed with precooled PBS and digested with trypsin digestion reagent. RIPA cell lysate was used to fully lysate the cells, and protein concentration was determined with BCA protein determination kit (Bio-Rad, Hercules, CA, USA). The protein was quantified to 1.5 *μ*g/mL by adding the loading buffer, protein, and PBS in proportion. After 2 h of 100 V electrophoresis, it was transferred to PVDF membrane. The proteins were blocked with blocking solution. The proteins were incubated with specific antibodies against HIF-1*α*, YAP, TAZ (Abcam, CA, USA), GAPDH (Proteintech, Rosemont, IL, USA) overnight, and then incubated with corresponding secondary antibody at room temperature for 2 h. ChemiScope Mini chemiluminescence instrument was used to detect and photograph the gray value of the target protein and calculate the expression of the target protein [24].

### 2.6. Migration Assay

Cell migration can be analyzed using the scratch healing test with a 6-well plate. The experimental items used in the scratch experiment were strictly sterilized; 1 mL cell suspension (1 × 105 cells/mL) was added to each well of the six-well plate and incubated at 37°C and 5% CO_2_. When the cells were all attached to the six-well plate (24 h), the cell migration was observed and evaluated at different time points 0, 24, and 48 h. Nikon inverted microscope ECLIPSE TS100 EPifucence microscope and NIS Elements AR 3.1 software were used to collect images and calculate the scratch area for statistics. The experiment was repeated three times.

### 2.7. Invasion Assay

Invasion assays were performed in 24-well plates using a polycarbonate membrane (Corning, NY, USA) chamber with a pore size of 8 *μ*m. Dilute 1 : 5 with 60 *μ*L of Matrigel (BD Sciences, San Jose, CA, USA) and serum-free DMEM culture solution and coat the membrane. The gel-coated Transwell chamber was placed at 37°C incubator for 2 h to form a matrix barrier. In the upper chamber, serum-free DMEM culture solution was added into to prepare cell suspension (1 × 105 cells/mL) of SiHa groups and C33a groups. 600 mL DMEM containing 10% fetal bovine serum was injected into the lower chamber. Laying the cells, the 24-well plate was incubated at 37°C incubator for 24 hours, and the invaded cells were washed with PBS cell buffer solution and fixed with 4% paraformaldehyde. Stain with crystal violet for 20 minutes. The cells remaining on top of the polycarbonate membrane were removed with a cotton swab. Images were taken under a Nikon ECLIPSE TS100 fluorescence microscope and the number of the cells was manually counted in 4 random areas.

### 2.8. MTT Assay

Cervical cancer cells SiHa, shNON, shHIF-1*α*, C33a, LV-NON, and LV-HIF-1*α* were at a density of 4000 cells/well in 96-well plates incubated overnight. After the cells were attached, at 0 h, 12 h, 24 h, 48 h, and72 h, 200 *μ*L of 0.5 mg/mL MTT 3-(4,5-dimethylthiazol-2-yl)-2,5-diphenyltetrazolium bromide was added. After 4 h of incubation, at 37°C, the MTT formazan precipitate was dissolved in 150 *μ*L of DMSO (Sigma, USA) to solubilize the formazan crystals, in a shaker before test, and the absorbance is at 490 nm using a microplate autoreader (Bio-Rad, USA).

### 2.9. Tumor Xenograft Mouse Model

The 4-week-old nude mice were randomly divided into 6 groups (6 mice/group) including SiHa, shNON, shHIF-1*α*, C33a, lev-non, and lev-HIF-1*α*. We subcutaneously injected 5 × 106 cell suspensions into each nude mouse. In animal experiments, the condition of tumor implantation in nude mice was observed weekly and the tumor volume was measured. 8 weeks after tumor implantation, all nude mice were anesthetized and euthanized with cervical dislocation. The weight and volume of the tumor were then measured and the results tallied.

### 2.10. Statistical Analyses

To ensure experimental reproducibility, all experiments were performed independently in triplicate, using GraphPad Prism 8.0 software (GraphPad software, Inc., La Jolla, CA, USA) to generate the presented statistical graphs and conducted statistical analysis of the differences. All statistics SPSS software (version 19.0) was used for analysis, and data were expressed as mean ± standard deviation (SD). The Mann-Whitney test was used to detect HIF-1*α* and YAP/TAZ immunohistochemical score between the cervical cancer group, CIN group, and normal tissue group. The relationship between the expression of HIF-1*α*, YAP/TAZ, and clinicopathological characteristics was detected by chi-square test or Fisher's exact test, and Spearman rank correlation analysis was used to analyze the correlation between HIF-1*α* and YAP/TAZ. ImageJ software calculates the scratch area and Transwell assay cell count, Image lab calculates the gray value of the Western blot band, and the result *p* < 0.05 was considered statistically significant.

## 3. Result

### 3.1. HIF-1*α* and YAP/TAZ Upregulated in Human Cervical Cancer Tissue

**Figure 1:**
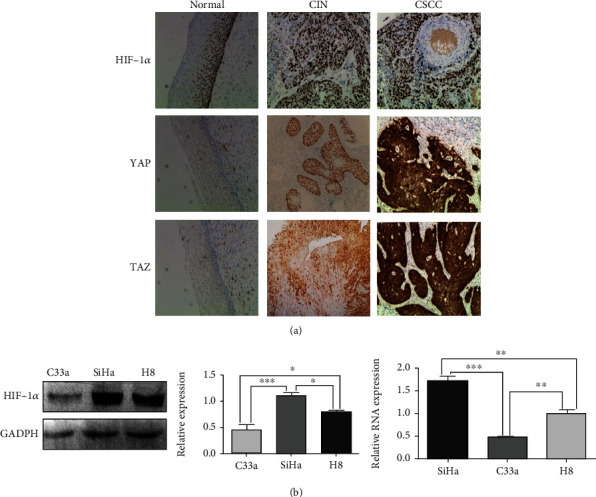
Expressions of HIF-1*α*, YAP, and TAZ in different pathological tissues and cell lines. (a) Expressions of HIF-1*α*, YAP, and TAZ in different pathological tissues. (b) The protein expression and mRNA level of HIF-1*α* in C33a and SiHa cells. ^∗^*p* < 0.05, ^∗∗^*p* < 0.01, and ^∗∗∗^*p* < 0.001.

The expression levels of HIF-1*α*, YAP, and TAZ in 30 normal cervical tissues, 58 CIN tissues, and 40 cervical squamous cell carcinoma tissues were detected by immunohistochemistry. The expression level of HIF-1*α* in cervical cancer tissue was higher than CIN and normal cervical tissue. HIF-1*α* expression was significantly increased in cervical squamous cell carcinoma compared with CIN and normal cervical tissue (Figure 1(a)). To further understand the clinicopathological significance of HIF-1*α* expression in cervical squamous cell carcinoma, we analyzed the relationship between HIF-1*α* expression and clinicopathological features of cervical squamous cell carcinoma (Table 1). The positive expression rates of HIF-1*α*, YAP, and TAZ in normal cervical tissue group were significantly lower than the CIN group and cervical squamous cell carcinoma group. The positive expression rates of HIF-1*α*, YAP, and TAZ in normal cervical tissue group were 20%, 23.3%, and 26.7%, respectively. The positive expression rates of CIN were 56.9%, 58.6%, and 62.1%, respectively. In CSCC, the positive expression rates of HIF-1*α*, YAP, and TAZ were 65.0%,72.5%, and 67.5%, respectively, with statistically significant differences (*p* < 0.05). The positive expression rate of HIF-1*α* in poorly differentiated group and lymph node metastasis group exceeds than that in highly differentiated and nonmetastasis groups, but the difference was not statistically significant (*p* > 0.05). YAP is related to the differentiation of CSCC. The positive expression rates of YAP protein in high differentiated and poorly differentiated CSCC were 57.9% and 85.7%, respectively, and statistically significant differences (*p* < 0.05). TAZ is correlated with node metastasis of CSCC. The positive expression rates of TAZ protein were 86.7% and 56.0%, respectively, in the group with and without lymph node metastasis, with statistically significant differences (*p* < 0.05). HIF-1*α* was positively related to YAP expression in CSCC (*r* = 0.487, *p* < 0.05). Increased expressions of HIF-1*α*, YAP, and TAZ in CSCC accelerate the development of cervical carcinoma. High expression of HIF-1*α*, YAP, and TAZ is of great significance for clinical prognosis.

To further understand whether the expression of HIF-1*α* in cervical squamous carcinoma cells is similar to that in tissue samples, we detected the expression of HIF-1*α* in SiHa, c33a, and normal cervical (H8) and the relationship between altered HIF-1*α* expression and YAP/TAZ expression. As expected, the expression level of HIF-1*α* was significantly higher in SiHa cells than in H8 cells (Figure 1(b)). Furthermore, the expression of HIF-1 was decreased in c33a cells compared with SiHa cells. The mRNA level of HIF-1 in cellular *α* was consistent with its protein level (Table 2). These results further support that the HIF-1*α* expression was upregulated in CC.

### 3.2. HIF-1*α*-Activated Hippo Pathway (YAP/TAZ) Gene Is Expressed in Cervical Cancer Cells

**Figure 2:**
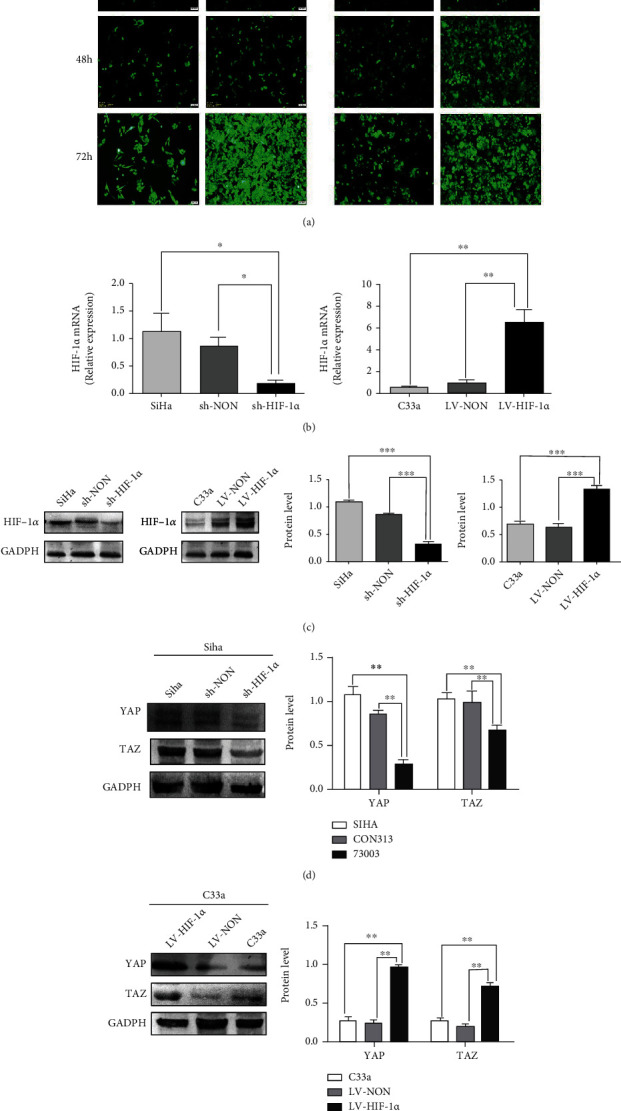
Knockdown of HIF-1 in SiHa cells and increase HIF-1 in C33a cells. (a) C33a and SiHa cells in fluorescence after transfection. (b) The result of qRT-PCR HIF-1*α* overexpression and downexpression in SiHa and C33a. (c) Western blot results of HIF-1*α* after downregulation and upregulation in SiHa and C33a. (d) The expression levels of YAP and TAZ in SiHa cells. (e) The expression levels of YAP and TAZ in C33a cells. ^∗^*p* < 0.05, ^∗∗^*p* < 0.01, and ^∗∗∗^*p* < 0.001.

To determine whether HIF-1*α* can regulate downstream genes of the Hippo pathway (YAP/TAZ) in cervical squamous cell carcinoma cells, we first constructed stable overexpressing HIF-1*α* C33a cells (LV-HIF-1*α*), which initially expressed HIF-1*α* at a relatively low level. Meanwhile, stable HIF-1*α*-knockout SiHa cells (Sh-HIF-1*α*) were constructed, which initially expressed high levels of HIF-1*α* (Figure 2(a)). We then detected the changes of HIF-1*α* mRNA after lentivirus transfection and found that lentivirus infection caused significant changes in HIF-1*α* mRNA (Figure 2(b)). Western blot confirmed that HIF-1*α* expression was successfully altered after lentivirus infection (Figure 2(c)). Subsequently, Western blotting results showed that (YAP/TAZ) expression was decreased in shHIF-1*α* cells (Figure 2(d)), while (YAP/TAZ) expression was significantly increased in LV-HIF-1*α* cells (Figure 2(e)). In conclusion, HIF-1*α* significantly affects the expression of YAP/TAZ in cervical squamous cell carcinoma cells, suggesting that HIF-1*α* can activate the expression of YAP/TAZ gene downstream of Hippo pathway in cervical cells.

### 3.3. Effects of HIF-1*α* on Migration and Invasion of CC Cells

**Figure 3:**
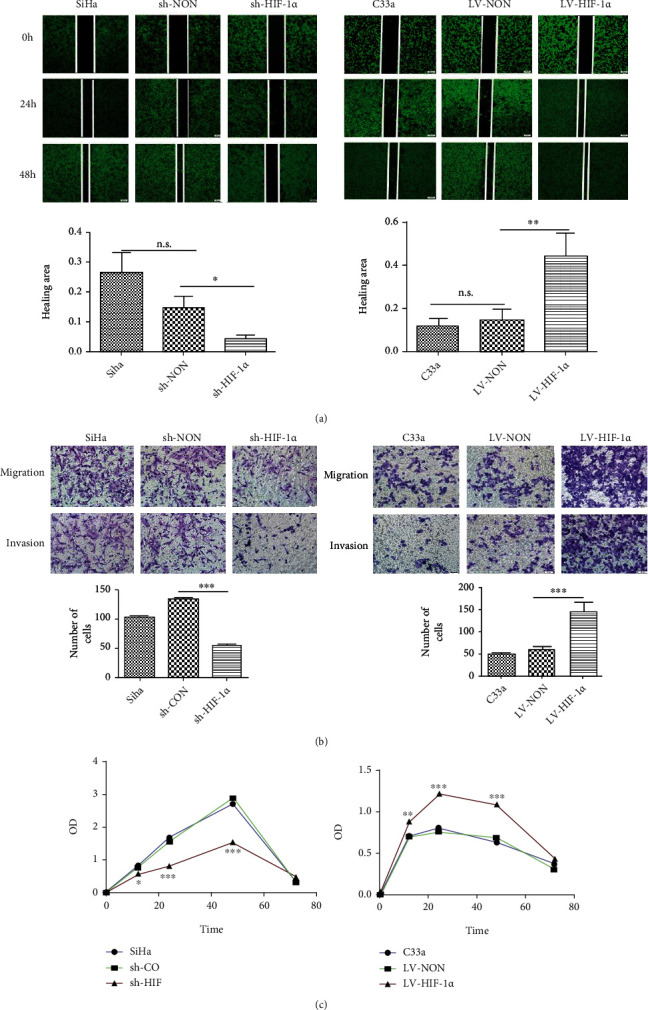
Effects of HIF-1*α* on migration and invasion of CC cells. (a) The result of migration capacity after changed HIF-1*α* expression (×100). (b) The result of invasive capacity after changed HIF-1*α* expression in SiHa (×100) and C33a (×100). (c) Changes of cell proliferation ability after knockdown and overexpression of HIF-1*α* in CSCC cells. ^∗^*p* < 0.05, ^∗∗^*p* < 0.01, and ^∗∗∗^*p* < 0.001.

To further investigate the role of HIF-1*α* in the migration, invasion, and proliferation of cervical squamous cell carcinoma cells, we performed wound healing assay, Transwell invasion assay, and MTT assay. HIF-1*α* knockdown resulted in a significant decrease in migration capacity in a time-dependent manner (Figure 3(a)). A significant reduction in the ability of cells to invade was also observed (Figure 3(b)). However, overexpression of HIF-1*α* significantly increased cell migration and invasion. These results suggest that HIF-1*α* may regulate the expression of YAP/TAZ and promote the invasion and metastasis of CC cells. At the same time, MTT assay results showed that upregulation of HIF-1*α* expression could significantly increase the proliferation of C33a cells, while downregulation of HIF-1*α* expression could significantly reduce the proliferation of SiHa cells (Figure 3(c)). These results suggested that HIF-1*α* can increase the proliferation rate of tumor cells.

### 3.4. The Effect of HIF-1*α* on Tumor Cell Proliferation In Vivo

**Figure 4:**
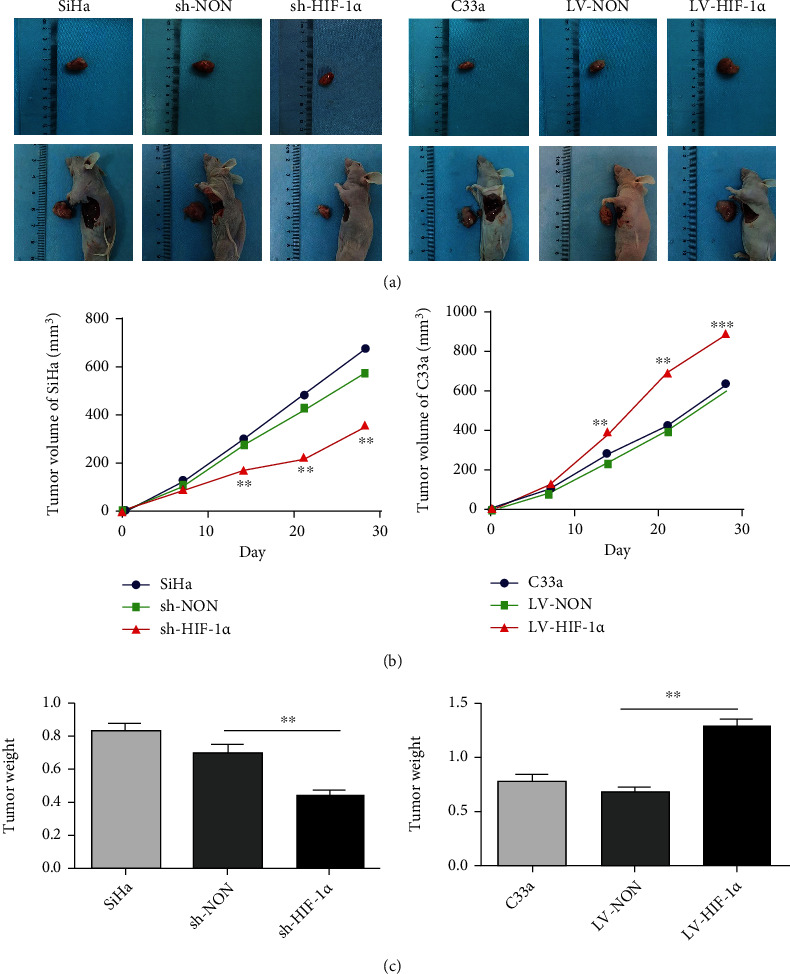
The effect of HIF-1*α* on tumor cell proliferation in vivo. (a) A representative image of an anatomical tumor after transplantation in a nude mouse. (b) The volume growth curves of subcutaneous tumors in different groups of mice were shown. (c) The average tumor weight of the animals in the experimental group when they were treated. ^∗∗^*p* < 0.01 and ^∗∗∗^*p* < 0.001.

Due to the gap between in vitro and in vivo experiments, to further confirm the effect of HIF-1*α* on tumor cell proliferation in vivo, we injected HIF-1*α* overexpressed C33a cells and HIF-1*α*-knockout SiHa cells into nude mice. Compared with control mice, tumors in mice with overexpressed HIF-1*α* C33a cells were significantly larger. In contrast, the tumors of nude mice transfected with HIF-1*α*-knockout SiHa cells were significantly smaller (Figure 4(a)). This indicates that HIF-1*α* can promote tumor proliferation in vivo. During this experiment, the nude mice were observed every other week and the size of the tumor was recorded. After 28 days, the nude mice were treated (euthanasia). The results showed that HIF-1*α* expression affected tumor proliferation. This is the volume of the tumor measured weekly (Figure 4(b)). Tumor weight measured weekly is shown (Figure 4(c)).

## 4. Discussion

HIF-1*α* is a key hypoxia-inducible factor subunit that mediates the adaptive response of cells in a hypoxic microenvironment. HIF-1*α* gene expression programs can help cells with energy metabolism, adapt to hypoxia stress, glycolysis, and metabolism, and control angiogenesis, erythropoiesis, tumor invasion, migration, and activation of protein genes related to tumor progression [25–27]. Studies have shown that upregulation of HIF-1*α* activity promotes tumor-associated angiogenesis, thereby promoting tumor cell survival and proliferation in solid tumors [28].

HIF-1*α* silencing inhibits the growth and invasion of cancer cells and induces an increased rate of apoptosis in vitro [29]. Compared with normal liver tissue, viral hepatitis, liver cirrhosis, and hepatocellular carcinoma are more obvious; in addition, HIF-1*α*-induced glucose transporter-1 (G-lut1) and vascular endothelial growth factor (VEGF) gene expressions promote glycolysis and induce angiogenesis, which is closely related to the occurrence, development, and proliferation of cancerous cells of liver cancer [30].

Hippo pathway core components (YAP/TAZ) are essential for tissue development and tissue homeostasis, and abnormal expression of Hippo pathway core components is involved in a variety of pathological diseases, including cancer [31].

YAP and TAZ are two key transcriptional coactivators downstream of the Hippo pathway [32]. Whether HIF-1*α* promotes tumor invasion and metastasis by activating YAP/TAZ is rarely reported. On the basis of our previous work, we used immunohistochemistry to detect the expression of HIF-1*α* and YAP/TAZ in normal cervical tissues, cervical intraepithelial neoplasia (CIN), and cervical cancer tissues. We analyzed the association between HIF-1*α*, YAP/TAZ, and clinical malignant phenotypes of cervical cancer, such as differentiation degree and lymph node metastasis, and analyzed the correlation between HIF-1*α*-positive expression and YAP/TAZ expression in cervical cancer tissues. The positive expression rates of HIF-1*α*, YAP, and TAZ proteins in normal cervical tissues were lower than those in CIN and CSCC. The positive expression rate of HIF-1*α* in low differentiation group and lymph node metastasis group was higher than that in high differentiation group and no lymph node metastasis group. The positive expression rate of YAP in well-differentiated squamous cell carcinoma was lower than that in poorly differentiated squamous cell carcinoma. TAZ was associated with CSCC lymph node metastasis, and the positive expression rate of TAZ protein in the group with lymph node metastasis was higher than that in the group without lymph node metastasis. HIF-1*α* was positively correlated with YAP protein expression in CSCC tissue.

The experimental results in this part of the study are basically consistent with the relevant reports, indicating that HIF-1*α* and YAP/TAZ have certain value in the diagnosis of cervical cancer and the judgment of malignant degree and prognosis.

The second part of the study mainly conducted in-depth research on cervical squamous cell carcinoma cells at the cell level in vitro, detected the expression level of HIF-1*α* in different cervical cancer cells, and analyzed the effect of HIF-1*α* expression level on the invasion and metastasis of cervical cancer cells. The effect of HIF-1*α* on the expression of Hippo signaling pathway target gene YAP/TAZ and the function of cervical cancer cells during the occurrence and development of cervical cancer.

Firstly, the expression of HIF-1*α* in cervical normal tissue cells H8, cervical squamous cell carcinoma SiHa (HPV+), and cervical squamous cell carcinoma C33a (HPV-) was detected by QRT-PCR and Western blot. The results showed that HIF-1*α* expression level in SiHa (HPV+) was significantly higher than that in normal cervical tissue cell H8 and cervical squamous cell C33a (HPV-). Therefore, we used lentivirus transfection technology to investigate the mechanism of HIF-1*α* in cervical cancer metastasis and the effect on Hippo pathway target and cervical cancer by cell functional level and molecular level. We transfected cervical squamous cell carcinoma cells with lentivirus for knockdown and overexpression of HIF-1*α* and screened out successfully transfected knockdown SiHa (HPV+) cells and overexpression C33A (HPV-) cells by flow cytometry screening. Then, the expression of HIF-1*α* in transfected cells was detected by QRT-PCR and Western blot. The results suggested that the mRNA and protein expression levels of HIF-1*α* in SH-HIF-1A group were significantly lower than those in the SH-NON group and control group. The mRNA and protein expression levels of HIF-1*α* in the LV-HIF-1*α* overexpression group were significantly higher than those in the LV-non group and control group. After successfully constructing the lentivirus vector model, we conducted experiments on cell function and molecular mechanism: (1) The changes of proliferation, invasion, and migration of cervical cancer cells and Hippo pathway-related protein YAP/TAZ after knockdown and overexpression of HIF-1*α* were detected. The results showed that knockdown of HIF-1*α* inhibited the proliferation, invasion, migration and scratch healing process of cervical cancer cells, while overexpression of HIF-1*α* promoted the proliferation, invasion, migration, and scratch healing process of cervical cancer cells, thus confirming that HIF-1*α* promoted tumor metastasis by regulating the invasion, migration and scratch healing process of cervical cancer cells. (2) The expression changes of YAP/TAZ downstream of Hippo pathway in cervical cancer cells after HIF-1*α* knockdown and overexpression were detected to understand the changes of invasion, migration, and proliferation of cervical cancer cells. The results showed that after HIF-1*α* knockdown, the expression of YAP/TAZ decreased, and the invasion, migration, and proliferation of cervical cancer cells were weakened. On the contrary, overexpression of HIF-1*α* increased the expression of YAP/TAZ, and the invasion, migration, and proliferation of cervical cancer cells were enhanced, suggesting that HIF-1*α* regulates the development of cervical cancer by regulating the YAP/TAZ target.

In the first two parts of this study, we confirmed the role of HIF-1*α* in the progression, invasion, and metastasis of cervical cancer from the tissue and cellular levels.

In the third part, on the basis of the previous in vitro test, cervical cancer cells with knockdown and overexpression of HIF-1*α* gene were established by lentivirus transfection and injected into the subcutaneous (armpit) of nude mice to observe the effect on tumor formation in nude mice. HIF-1*α* was downregulated in SiHa and upregulated in C33a and then injected into the nude mice to obtain subcutaneous transplanted tumors. The volume and weight of transplanted tumor were measured to evaluate the effect of HIF-1*α* gene on tumor formation in nude mice. To understand the effect of HIF-1*α* on the proliferation of cervical cancer in vivo, this method first ensures the unity of the genetic background of tumor tissue, and secondly, this method has a short test period, good reproducibility, and simple operation. The volume and weight of tumor formation in vivo were significantly larger than those of the nude mice in the knockout group. This indicates that the upregulation of HIF-1*α* expression has a promoting effect on tumor growth in vivo. This result is consistent with the results of in vitro experiments, which further proves that the gene HIF-1*α* exists as an oncogene in cervical cancer. In summary, HIF-1*α* plays an important role in the growth, invasion, and metastasis of cervical cancer cells, and its mechanism may be involved in the occurrence and development of cervical cancer through its downstream tumor microenvironment-related proteins. We speculate that HIF-1*α* gene silencing inhibits the growth and metastasis of cervical cancer cells through a variety of ways and may become a potential effective target for cervical cancer gene therapy.

## Figures and Tables

**Table 1:** According to the histopathological characteristics of the patient and expression of HIF-1*α*, YAP, and TAZ in CC.

Groups	*N*	HIF-1		*χ* ^2^	*p*	YAP		*χ* ^2^	*p*	TAZ		*χ* ^2^	*p*
		Positive	Negative			Positive	Negative			Positive	Negative		
A (normal cervical epithelial)	30	6 (20.0)	24 (80.0)			7 (23.3)	23 (76.7)			8 (26.7)	22 (73.3)		
B (CIN)	58	33 (56.9)	25 (43.1)			34 (58.6)	24 (41.4)			36 (62.1)	22 (37.9)		
B1 (CINI)	32	15 (46.9)	17 (53.1)			16 (50.0)	16 (50.0)			17 (53.1)	15 (46.9)		
B2 (CINII-III)	26	18 (69.2)	8 (30.8)			18 (69.2)	8 (30.8)			19 (73.1)	7 (26.9)		
C (CSCC)	40	26 (65.0)	14 (35.0)	15.48	0.001	29 (72.5)	11 (27.5)	17.39	0.001	27 (67.5)	13 (32.5)	13.44	0.001
Differentiation													
C1 (high)	19	10 (52.6)	9 (47.4)			11 (57.9)	8 (42.1)			12 (63.2)	7 (36.8)		
C2 (moderate/poor)	21	16 (76.1)	5 (23.9)	2.43	0.12	18 (85.7)	3 (14.3)	3.87	0.049	15 (71.4)	6 (28.6)	0.31	0.577
L/N metastasis													
C3 (positive)	15	11 (73.3)	4 (26.7)			12 (80.0)	3 (20.0)			13 (86.7)	2 (13.3)		
C4 (negative)	25	15 (60.0)	10 (40.0)	0.73	0.39	17 (68.0)	8 (32.0)	0.68	0.411	14 (56.0)	11 (44.0)	4.02	0.045

**Table 2:** HIF-1*α* mRNA and protein expression levels in C33a, SiHa, and H8.

Group	HIF-1*α* mRNA	HIF-1*α* pro
SiHa	1.7268 ± 0.8743	1.1044 ± 0.0911
C33a	0.4764 ± 0.3471	0.5869 ± 0.0059
H8	0.8811 ± 0.1553	0.6812 ± 0.0618
*F*	66.36	24.82
*p*	<0.0001	0.0013

## Data Availability

The data used to support the findings of this study are included within the article.

## Abbreviations

CCSC: Cervical squamous cell carcinoma
CIN: Cervical intraepithelial neoplasia
HIF-1*α*: Hypoxia-inducible factor 1
YAP: Yes-related protein
TAZ: Transcriptional coactivator with PDZ binding motif.
